# 2-Methoxyestradiol Induces Vasodilation by Stimulating NO Release via PPARγ/PI3K/Akt Pathway

**DOI:** 10.1371/journal.pone.0118902

**Published:** 2015-03-06

**Authors:** Weiyu Chen, Yuhong Cui, Shuhui Zheng, Jinghe Huang, Ping Li, Tommaso Simoncini, Yongfu Zhang, Xiaodong Fu

**Affiliations:** 1 School of Basic Sciences, Guangzhou Medical University, Guangzhou, 510182, Guangdong Province, China; 2 Department of Physiology, Zhongshan School of Medicine, Sun Yat-sen University, Guangzhou, 510080, Guangdong Province, China; 3 Research Center of Translational Medicine, the First Affiliated Hospital, Sun Yat-Sen University, Guangzhou, 510080, Guangdong Province, China; 4 Molecular and Cellular Gynecological Endocrinology Laboratory (MCGEL), Department of Reproductive Medicine and Child Development, University of Pisa, Pisa, 56100, Italy; 5 Department of Anesthesiology, Guangzhou Women and Children's Medical Center, Guangzhou, 510180, Guangdong Province, China; University of Pittsburgh, UNITED STATES

## Abstract

The endogenous estradiol metabolite 2-methoxyestradiol (2-ME) reduces atherosclerotic lesion formation, while the underlying mechanisms remain obscure. In this work, we investigated the vasodilatory effect of 2-ME and the role of nitric oxide (NO) involved. *In vivo* studies using noninvasive tail-cuff methods showed that 2-ME decreased blood pressure in Sprague Dawley rats. Furthermore, *in vitro* studies showed that cumulative addition of 2-ME to the aorta caused a dose- and endothelium-dependent vasodilation. This effect was unaffected by the pretreatment with the pure estrogen receptor antagonist ICI 182,780, but was largely impaired by endothelial nitric oxide synthase (eNOS) inhibitor NG-nitro-L-arginine methyl ester (L-NAME) or by phosphoinositide 3-kinase (PI3K) inhibitor wortmannin (WM). Moreover, 2-ME（10^−7 ∼^10^−5^ M）enhanced phosphorylation of Akt and eNOS and promoted NO release from cultured human umbilical endothelial cells (HUVECs). These effects were blocked by PI3K inhibitor WM, or by the transfection with Akt specific siRNA, indicating that endothelial Akt/eNOS/NO cascade plays a crucial role in 2-ME-induced vasodilation. The peroxisome proliferator-activated receptor γ (PPARγ) mRNA and protein expression were detected in HUVECs and the antagonist GW9662 or the transfection with specific PPARγ siRNA inhibited 2-ME-induced eNOS and Akt phosphorylation, leading to the impairment of NO production and vasodilation. In conclusion, 2-ME induces vasodilation by stimulating NO release. These actions may be mediated by PPARγ and the subsequent activation of Akt/eNOS cascade in vascular endothelial cells.

## Introduction

Cardiovascular disease is the most common cause of death in women. Estrogen is believed to provide cardiovascular protective effects. However, no overall cardiovascular benefit was found in postmenopausal women receiving conjugated equine estrogen in WHI trial [1]. Thus, a beneficial effect of estrogen on cardiovascular disease is still an open question and novel strategies should be developed to protect postmenopausal women against cardiovascular disease.

Recent years emerging evidence suggests that 2-methoxyestradiol (2-ME) may represent a valuable therapeutic molecule for prevention of cardiovascular disease [2]. 2-ME derives from the NADPH-dependent cytochrome P450 metabolism of 17β-estradiol (E2) and it lacks affinity for estrogen receptors [3]. In addition to its potent anticarcinogenic properties, 2-ME shares similar actions as E2 in the cardiovascular system. For example, it attenuates hypertension and coronary vascular remodeling [4], reduces atherosclerotic lesion formation [5] and inhibits neointima formation [6]. These cardiovascular effects are mainly attributed to the actions of 2-ME on vascular smooth muscle cells (VSMCs) [7], while the actions of 2-ME on vascular endothelial cells remain obscure.

Endothelial dysfunction is the first step towards cardiovascular disease such as atherosclerosis [8]. It is characterized by impaired vasodilation, pro-thrombosis, pro-coagulation, etc [9]. Our previous work has demonstrated that endothelial nitric oxide synthase (eNOS)/ nitric oxide (NO) plays a crucial role in E2-induced vasodilation [10,11]. Likewise, it has been reported that 2-ME potentiated the vasodilatory effect of A23187 on rat intestinal arterioles by stimulating endothelial NO release [12]. However, the direct effects of 2-ME on vasodilation and the underlying mechanism responsible for NO production are still the matter of investigation.

In this study, we investigated the effect of 2-ME on vasodilation and the role of Akt/eNOS/NO cascade involved. Moreover, we explored the role of peroxisome proliferator-activated receptor γ (PPARγ) in regulating these signaling pathways in vascular endothelial cells.

## Results

### 2-ME Decreased Blood Pressure in Sprague Dawley Rats

**Fig 1:**
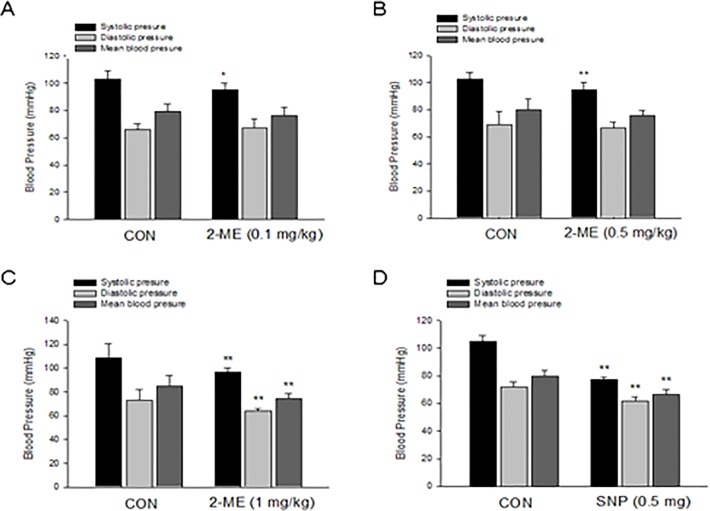
2-ME decreases blood pressure in Sprague Dawley rats. A-D. Noninvasive tail-cuff method was used to measure the systolic, diastolic and mean blood pressure in Sprague Dawley (SD) rats. Rats were received peritoneal injection of 2-ME at low concentration (0.1 mg/kg body weight) (A), moderate concentration (0.5 mg/kg body weight) (B), high concentration (1 mg/kg body weight) (C) or of sodium nitroprussiate (0.5 mg/mL, 1 mL) (D) for 20 min and then blood pressure was recorded. n = 5. * P < 0.05 vs corresponding control, P < 0.01 vs corresponding control.

To observe the overall effect of 2-ME on vasodilation, we used noninvasive tail-cuff methods to measure the systolic, diastolic and mean blood pressure in Sprague Dawley (SD) rats. Treatment with 2-ME at a low or moderate concentration (0.1 mg/kg or 0.5 mg/kg body weight) for 20 min both led to a significant decrease of systolic pressure (SP) when compared with that before peritoneal injection, while there was no difference for diastolic or mean blood pressure (Fig. 1A, 1B). The high concentration of 2-ME (1 mg/kg body weight), however, resulted in significant decrease of systolic, diastolic and mean blood pressure in SD rats (Fig. 1C). As a positive control, injection of sodium nitroprussiate (0.5 mg/mL, 1 mL) greatly reduced rat blood pressure (Fig. 1D). Meanwhile, injection of physiological saline (1 mL) had no effect on rat blood pressure.

### 2-ME Caused a Dose- and Endothelium-Dependent Relaxation

**Fig 2:**
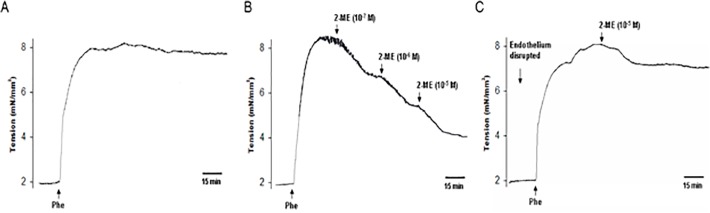
2-ME causes a dose- and endothelium-dependent relaxation. A. Aorta was preconstricted with phenylephrine (Phe, 100 nM) and wall tension was recorded. B. Representative changes in wall tensions of rat aorta induced by a cumulative application of 2-ME in concentrations from 10^−7^ to 10^−5^ M. All arteries were preconstricted with Phe (100 nM) before testing with 2-ME. C. Endothelial cell layer was disrupted with a stainless steel wire. Then arteries were preconstricted with Phe (100 nM) before testing with 2-ME (10^−5^ M). All the experiments were repeated six times with consistent results.

Aortas were preconstricted by using phenylephrin (Phe, 100 nmol/L) and the stable contraction was achieved and it lasted for more than 2 h (Fig. 2A). After reaching the plateau, 2-ME was added in order to induce vasorelaxation. Cumulative addition of 2-ME from 10^−7^ to 10^−5^ mol/L caused a dose-dependent relaxation. The magnitude of relaxation was expressed as percentage of the maximal contraction induced by Phe. As shown in Fig. 2B, 2-ME (10^−7^ mol/L) induced aorta relaxation at a magnitude of (43.0 ± 4.8)% (n = 6, P < 0.01). After this vasodilatory effect reached the maximal, 2-ME (10^−6^ mol/L) was added and it further led to relaxation, with the magnitude of (62.3 ± 5.5)% (n = 6, P < 0.01). Likewise, the magnitude of dilation was potentiated by the addition of 10^−5^ mol/L 2-ME ((73.4 ± 5.0)%) (n = 6, P < 0.01).

To test whether 2-ME-induced vasorelaxation is endothelium-dependent, endothelial cell layer was disrupted with a stainless steel wire. The endothelium was considered to be functionally disrupted if the relaxation produced by acetylcholine (ACh, 100 μmol/L) was decreased by at least 80% of the control response. As shown in Fig. 2C, when the endothelial cell layer was removed, 2-ME (10^−5^ mol/L) only induced a slight relaxation with the magnitude of (12.2 ± 5.1) % (n = 6, P < 0.05), suggesting that 2-ME-induced vasorelaxation was largely endothelium-dependent.

### The role of eNOS and PI3K in 2-ME-Induced Vasodilation

**Fig 3:**
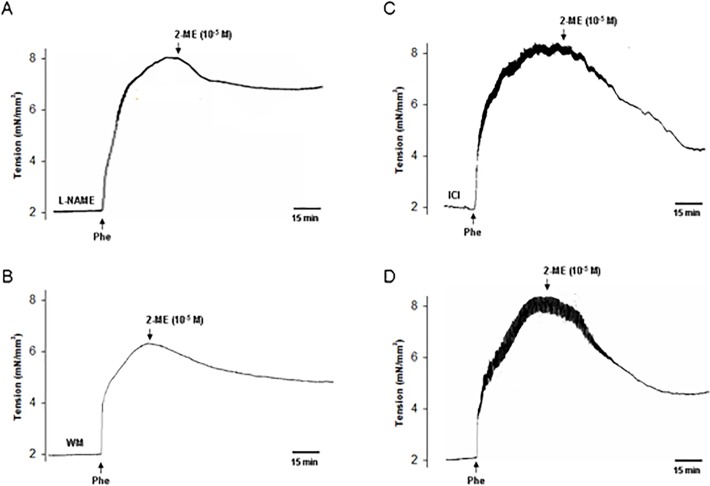
eNOS and PI3K are involved in 2-ME-induced vasodilation. A-D: Rat aortas pretreated with L-NAME (200 μM, A), wortmannin (WM—30 nM, B), ICI 182,780 (ICI -100 nM, C) or without any inhibitor (D) and their effects on 2-ME (10^−5^ M)-induced dilation were shown. All the experiments were repeated six times with consistent results.

NO functions as a key endothelial-derived vasodilator molecule. To determine its role in 2-ME-induced vasodilation, aorta rings were pretreated with the eNOS inhibitor L-NAME for 30 min and then the vasodilatory effect of 2-ME was observed. As a result, pretreatment with L-NAME largely inhibited 2-ME-induced vasodilation, with an inhibitory rate of (61.5 ± 6.8)% (n = 6, P < 0.01) (Fig. 3A).

PI3K/Akt pathway is the well-known upstream of eNOS. As shown in Fig. 3B, in the presence of PI3K inhibitor wortmannin (WM), the magnitude of 2-ME-induced vasodilation was substantially decreased. The inhibitory rate was (68.4 ± 7.5)% when compared with 2-ME treatment alone group (n = 6, P < 0.01).

Furthermore, to preclude the role of estrogen receptor (ER), the pure ER antagonist ICI 182,780 (ICI) was used and it didn’t change 2-ME-induced vasodilation (Fig. 3C). As a control, in the absence of these inhibitors, 2-ME (10^−5^ mol/L) led to the vasodilation (Fig. 3D).

### 2-ME Enhanced NO Release via PI3K/Akt Pathway in Human Endothelial Cells

**Fig 4:**
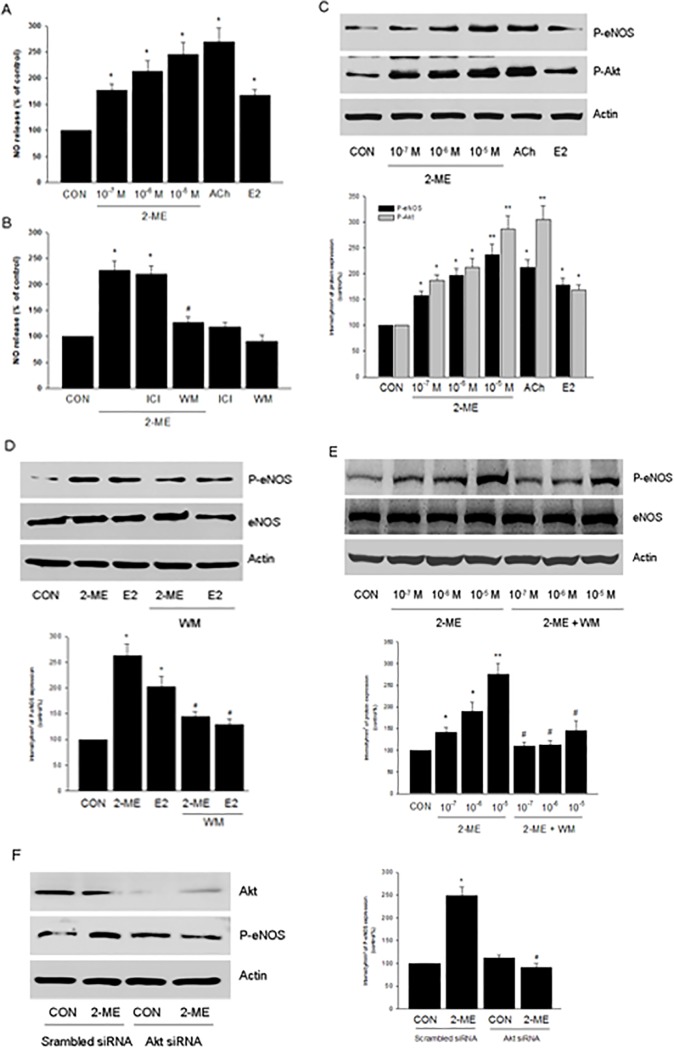
2-ME enhances NO release via PI3K/Akt pathway in human endothelial cells. A. HUVECs were exposed to 2-ME (10^−7^–10^−5^ M), ACh (10^−6^ mol/L) or E2 (10^−8^ mol/L) for 20 min. The medium NO concentrations were measured. n = 5, * P < 0.01 vs. CON. B. HUVECs were exposed to 2-ME (10^−5^ M) for 20 min, in the presence or absence of ICI 182,780 (ICI—100 nM) or wortmannin (WM—30 nM). n = 5, * P < 0.01 vs. CON; # P < 0.01 vs. 2-ME group. C. HUVECs were exposed to 2-ME (10^−7^–10^−5^ M), ACh (10^−6^ mol/L) or E2 (10^−8^ mol/L) for 20 min and cell content of phospho-eNOS (P-eNOS), phospho-Akt (P-Akt) or β-actin were shown by western blot. n = 3, * P < 0.01 vs. CON; ** P < 0.001 vs. CON. D. HUVECs were exposed to 2-ME (10^−5^ M) or E2 (10^−8^ mol/L) for 20 min, in the presence or absence of wortmannin (WM—30 nM). Cell content of phospho-eNOS (P-eNOS), eNOS or β-actin were shown by western blot. n = 3, * P < 0.01 vs. CON; # P < 0.01 vs. corresponding 2-ME or E2 group, respectively. E. HUVECs were exposed to 2-ME at different concentrations (10^−7^–10^−5^ M) for 20 min, in the presence or absence of WM. Cell content of phospho-eNOS (P-eNOS), eNOS or β-actin were shown by western blot. n = 3, * P < 0.01 vs. CON; ** P < 0.001 vs. CON; # P < 0.01 vs. corresponding 2-ME group, respectively. F. HUVECs were transfected with 100 nM target siRNAs for Akt or scrambled siRNA for 48 h and then treated with 2-ME (10^−5^ M) for 20 min. Cell content of Akt, phospho-eNOS (P-eNOS), or β-actin were shown by western blot. n = 3, * P < 0.01 vs. CON; # P < 0.01 vs. 2-ME transfected with scrambled siRNA.

In cultured HUVECs, treatment with 2-ME (10^−7 ∼^ 10^−5^ mol/L) for 20 min enhanced NO release in a dose-dependent manner (Fig. 4A). As positive controls, ACh (10^−6^ mol/L) or E2 (10^−8^ mol/L) also resulted in elevated NO release (Fig. 4A). The effect of 2-ME on NO release was inhibited by the addition of WM, but not by the addition of ICI (Fig. 4B).

Ser1177 phosphorylation represents eNOS activation. Indeed, 2-ME (10^−7 ∼^ 10^−5^ mol/L) all led to elevated level of eNOS phosphorylation at Ser1177 (Fig. 4C). Similarly, 2-ME increased Akt phosphorylation (Fig. 4C). eNOS activation induced by different dose of 2-ME was inhibited by the PI3K inhibitor WM (Fig. 4D and 4E), suggesting that PI3K/Akt plays a crucial role in eNOS activation and NO release. In line with this, silencing of Akt protein expression largely blocked 2-ME-enhanced eNOS phosphorylation (Fig. 4F).

### 2-ME activated Akt/eNOS via PPARγ in Human Endothelial Cells

**Fig 5:**
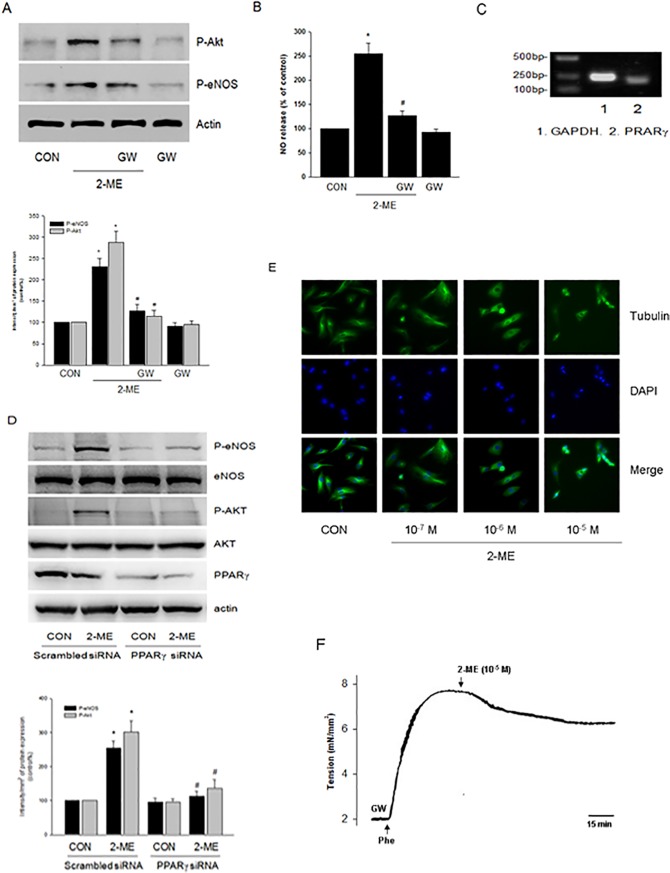
2-ME stimulates Akt/eNOS activation via PPARγ in endothelial cells. A. HUVECs were exposed to 2-ME (10^−5^ M) for 20 min, in the presence or absence of GW9662 (GW—20 μM). Cell content of phosphor-Akt (P-Akt), phospho-eNOS (P-eNOS) or β-actin were shown by western blot. n = 3, * P < 0.01 vs. CON; # P < 0.01 vs.2-ME. B. HUVECs were exposed to 2-ME (10^−5^ M) for 20 min, in the presence or absence of GW9662 (GW—20 μM). The medium NO concentrations were measured. n = 5, * P < 0.01 vs. CON; # P < 0.01 vs.2-ME. C. Expression of PPARγ mRNA was detected by RT-PCR using total RNA extracted from HUVECs. GAPDH mRNA was used as an internal control. D. HUVECs were transfected with 100 nM target siRNAs for PPARγ or scrambled siRNA for 48 h and then treated with 2-ME (10^−5^ M) for 20 min. Cell content of P-eNOS, eNOS, P-Akt, Akt, PPARγ or β-actin were shown by western blot. n = 3, * P < 0.01 vs. corresponding CON; # P < 0.01 vs. corresponding 2-ME treatment group transfected with scrambled siRNA. E. HUVECs were treated with 2-ME at different doses as indicated for 20 min and then cells were stained with an Ab vs. tubulin (FITC; green staining). Nuclei were counterstained in blue with DAPI. All the experiments were repeated three times with consistent results, and a representative result is shown.F. The effect of GW9662 (GW—20 μM) on 2-ME (10^−5^ M)-induced dilation were shown. The experiments were repeated six times with consistent results.

It was reported that 2-ME activates peroxisome proliferator-activated receptor γ (PPARγ) in vascular smooth muscle cells [7]. Therefore, we investigated the role of PPARγ in 2-ME-induced Akt/eNOS activation. As shown in Fig. 5A, PPARγ antagonist GW9662 largely inhibited 2-ME-enhanced eNOS and Akt phosphorylation. Accordingly, GW9662 impaired 2-ME-enhanced NO production (Fig. 5B).

To confirm the expression of PPARγ in human endothelial cells, we performed Reverse Transcription-Polymerase Chain Reaction (RT-PCR) to detect its mRNA expression and we detected the expected size of PPARγ fragments was 208 bp (Fig. 5C). In line with this, PPARγ protein expression was detected in HUVECs by western blot method (Fig. 5D).

To further confirm the role of PPARγ, we used specific siRNA to silence its protein expression and then we observed its influence on Akt/eNOS phosphorylation. Likewise, silencing of PPARγ blocked 2-ME-stimulated Akt and eNOS activation (Fig. 5D).

2-ME is known to interfere with the dynamics of tubulin polymerization [6]. In our study, when HUVECs were treated with 2-ME at a dose of 10^−7^ M for 20 min, the tubulin polymerization process was not altered, as indicated by immunofluorescence results (Fig. 5E). At higher concentrations (10^−6^ and 10^−5^ M), however, 2-ME disturbed tubulin polymerization to some extent (Fig. 5E).

To further confirm the role of PPARγ, we observed the effect of GW9662 on vasodilation. As a result, pretreatment with GW9662 largely impaired 2-ME-induced vasorelaxation, with an inhibitory rate of (69.2 ± 7.4)% (n = 6, P < 0.01) (Fig. 5F).

## Discussion

Estrogen replacement therapy is highly effective in the prevention of chronic diseases such as atherosclerosis and osteoporosis, while it increases the risk of developing breast and endometrial cancers [13]. Therefore, it is necessary to develop alternative agents that are safe and effective enough to improve the life qualities of postmenopausal women.

2-ME is a major endogenous metabolite of E2. It is formed via the sequential conversion of E2 to 2-hydroxyestradiol by cytochrome P450s (CYP450s) and then to 2-ME by catechol-O-methyltransferase (COMT) [2]. The valuable advantages of 2-ME over E2 are that it not only provides cardiovascular protective effects and may prevent osteoporosis, but also exerts anti-carcinogenic actions [2,14]. In cardiovascular system, 2-ME reportedly inhibits cell-cycle regulatory genes and proteins such as cyclin D1 and cyclin B1 in VSMCs [6,7]. To be noted, although the aberrant VSMCs proliferation and migration accelerates the progression of atherosclerosis, it occurs just in the middle or late stage, while endothelial dysfunction is the initial step in the development of atherosclerosis [15]. Currently, the actions and molecular mechanisms of 2-ME on vascular endothelial cells remain largely unknown. Our present work indicates that 2-ME induces vasodilation by increasing NO release via activation of PI3K/Akt/eNOS cascade. This effect is possibly mediated by PPARγ. These findings provide a new mechanistic explanation for understanding cardiovascular actions of 2-ME.

2-ME attenuates hypertension in spontaneously hypertensive rats [4]. Moreover, pregnant mice deficient in COMT show a pre-eclampsia-like phenotype resulting from an absence of 2-ME [16]. These studies indicate that 2-ME plays an important role in regulating vascular tone. This is confirmed in our work showing that 2-ME decreases blood pressure in SD rats. Previously, it was reported that pretreatment with 2-ME inhibited smooth muscle contractility through an endothelium- and NO-dependent mechanism. However, in that work, the application of 2-ME to Phe-preconstricted aorta didn’t induce significant relaxation [17]. More recently, Fenoy *et al* reported that 2-ME increased basal aortic endothelial NO production in male and in ovariectomized rats, while it had no effects on intact female rats [12]. These data are somewhat opposite to our observation that 2-ME stimulates NO release and leads to vasodilation in Phe-preconstricted female rat aorta. Furthermore, in our study, 2-ME rapidly induces vasodilation and stimulates endothelial NO release, suggesting that these effects maybe nongenomic. Indeed, 2-ME demonstrated a nongenomic inhibition of agonist-induced extracellular Ca^2+^-dependent contraction since it was not influenced by the protein synthesis inhibitor cycloheximide [18]. On the contrary, it was shown that inhibition of rat aortic smooth muscle contraction by 2-ME involved de novo protein synthesis [17]. The reason for these apparent discrepancies is unclear and it could be due to different experimental conditions or arterial preparations used in the studies. This, however, needs to be further investigated.

As mentioned above, 2-ME inhibits vascular contractility via NO release. However, no detailed mechanism has been verified. To our knowledge, this is the first time showing that 2-ME induces eNOS phosophorylation and NO release in cultured human endothelial cells. Notwithstanding, the inhibition of NO production by L-NAME couldn’t completely abolish 2-ME-induced vasodilation, implying that other mechanisms may also be involved. It is well known that NO, prostacylin and hydrogen sulfide (H_2_S) are three major vasodilatory molecules [19]. Seeger *et al* reported that 2-ME induced endothelial COX-2 expression, leading to prostacyclin production [20]. In addition, we have discovered that E2 activated H_2_S release in endothelial cells [21]. Therefore, the role of these molecules in 2-ME actions should be further explored.

It is well recognized that PI3K/Akt is the upstream signaling of eNOS. For example, we have shown that E2 stimulated endothelial NO release via PI3K/Akt activation [10]. In this work, we confirmed that 2-ME stimulated eNOS phosphorylation via activation of PI3K/Akt. Interestingly, 2-ME inhibited PI3K/Akt pathway in cancer cells and in vascular smooth muscle cells [6,22,23]. These observations, together with ours, suggest that 2-ME modulates PI3K/Akt activity in a tissue-specific manner.

The receptors that mediate the biological effects of 2-ME remain unidentified. 2-ME has no affinity for classical estrogen receptors (ERs) [24]. This was further confirmed by our observation that ICI 182,780 had no effect on its endothelial actions. 2-ME binds to tubulin at a relatively high concentration and its actions may partly due to the inhibition of tubulin polymerization [6,24]. However, in our study, the low concentration of 2-ME (10^−7^ M), which didn't affect tubulin polymerization as reported [6], also induced NO release and vasodilation, indicating that they were unlikely mediated by tubulin.

2-ME has structural similarities with PPARγ agonist rosiglitazone and it mimics the effects of rosiglitazone in vascular smooth muscle cells via a PPARγ-linked mechanism [6]. PPARγ is a ligand-activated transcription factor of the nuclear hormone receptor family and the activation of PPARγ improves vascular endothelial function, prevents the progression of intima-media thickening and atherosclerosis [25]. In particular, PPARγ regulates eNOS expression and phosphorylation via multiple mechanisms, including PI3K/Akt pathway [25]. In our study, we detected the abundant of PPARγ mRNA and protein expressions in HUVECs. The PPARγ antagonist GW9662 or the specific siRNA largely inhibited 2-ME-enhanced Akt and eNOS phosphorylation, suggesting that it regulated Akt/eNOS activity to induce endothelial NO release and vasodilation. However, it was reported that 2-ME had little affinity for PPARγ [6], implying that 2-ME may modulate PPARγ signaling via indirect mechanisms.

In our study, we focused on the cardiovascular effects of 2-ME in female. However, 2-ME also elicits beneficial effects in male. For instance, 2-ME stimulated NO release and vasodilation in male rats [12] and protected the vasculature from hypertension-induced myocardial arterial remodeling in male spontaneously hypertensive rats [4]. Moreover, 2-ME confered neuroprotection after traumatic brain injury in male mice [26] and had the potential of enhancing the antitumor effect on prostate cancer [27]. These evidence indicate that 2-ME may also be a promising therapeutic drug for the prevention of cardiovascular diseases in aged men.

In conclusion, our findings indicate that 2-ME induces vasodilation by stimulating endothelial NO release. These actions may be mediated by PPARγ and the subsequent activation of Akt/eNOS cascade in vascular endothelial cells. These findings will be helpful for better understanding the mechanisms through which 2-ME improves endothelial function and for better defining its potential use as an alternative agent in postmenopausal women.

## Materials and Methods

### Materials

2-methoxyestradiol (2-ME), 17β-estradiol (E2), NG-nitro-L-arginine methyl ester (L-NAME), phenylephrine (Phe), acetylcholine (ACh) and wortmannin (WM) were purchased from Sigma-Aldrich (St. Louis, MO, USA). ICI 182 780 was purchased from Tocris Cookson (Bristol, UK). Dulbecco’s modified Eagle’s medium (DMEM), Opti-MEM and fetal bovine serum (FBS) were obtained from Invitrogen (Carlsbad, CA, USA). All other chemicals were of analytical grade and purchased from Guangzhou Chemical Reagents (Guangzhou, China).

### Aorta Preparation and Wall Tension Measurement

*In vitro* tension measurement was carried out as we previously reported [21]. The animal experiments were conducted according to the guidelines of the National Research Council. Protocols were reviewed and consented by the Ethics Committee of Guangzhou Medical University. 10–11-week old Female Sprague-Dawley rats were obtained from the Center of Experimental Animals at Guangzhou Medical University (Guangzhou, China). All rats were housed under standard housing conditions and a 12 hour light-dark cycle. Food and water were available *ad libitum*. The rats were sacrificed by cervical dislocation. Aortas were collected from a region 1.5 cm left of the aortic arch and cut into four 2-mm wide ring segments in ice-cold physiological salt solution (PSS with the following composition (mmol/L): NaCl 119, KCl 4.7, CaCl_2_ 2.5, MgSO_4_ 1.17, NaHCO_3_ 25, KH_2_PO_4_ 1.18, EDTA 0.026 and glucose 5.5). To test whether the vasorelaxation is endothelium-dependent, endothelium was disrupted with a stainless steel wire. The rings were mounted on a two-chamber Danish Myotechnology M610 wire myograph under a stereomicroscope. The organ bath was filled with oxygenated Krebs solution and maintained at 37°C. Isometric contractions were recorded by a force transducer connected to an analog-to-digital converter system. Thirty minutes after mounting in the organ bath, all the rings were contracted using phenylephrine (Phe—100 nmol/L), and the functional integrity (over 80% relaxation) of the endothelial layer was determined by adding acetylcholine (ACh-100 μmol/L). The rings were then allowed to equilibrate for an additional 60 min and the rings were contracted for a second time by the addition of Phe for 10–15 min and then its response to 2-ME or E2 was recorded. For all the functional experiments, the rings were first incubated for 30 min with inhibitors.

### Blood Pressure Measurements: Noninvasive Tail-Cuff Methods

Experiments were performed in adult female Sprague Dawley rats (200–250 g). Rats were housed in individual cages and were fed *ad libitum* a normal chow diet for 1 week. Systolic blood pressure (SBP), diastolic blood pressure (DBP), and mean blood pressure (MBP) values were recorded in conscious, resting animals by noninvasive tail-cuff plethysmography (BP-100A, Taimeng Corporation, Chengdu, China) as previously described [28]. In brief, after a 3h fast, rats were placed in plastic restrainers which were maintained at 33–34°C. A cuff with a pneumatic pulse sensor was attached to the tail. On inflation, the cuff stopped blood flow through the tail, and on deflation the return of blood flow was detected by the sensor. Blood pressure values were recorded on BP Processing Software System (Taimeng Corporation, Chengdu, China) and were averaged from at least three consecutive readings obtained from each rat. After measuring basic blood pressure as control, rats received peritoneal injection of either physiological saline (1 mL, as negative control, n = 5), or different doses of 2-ME (1 mg/kg, 0.5 mg/kg, 0.1 mg/kg body weight, first dissolved in dimethyl sulphoxide with the parent solution concentration of 50 mg/ml, then dissolved in physiological saline for use, n = 5 for each concentration) or sodium nitroprussiate (0.5 mg, dissolved in physiological saline as positive control, n = 5). After injection of these substances for 20 minutes, the blood pressures were recorded and used for comparisons.

### Cell Cultures

Human umbilical vein endothelial cells (HUVECs) obtained from PromoCell (Heidelberg, Germany) were cultured as we previously described [29]. Before treatments, HUVECs were kept 24 hours in DMEM containing steroid-deprived FBS. Whenever an inhibitor was used, the compound was added 30 minutes before starting the treatments.

### Reverse Transcription-Polymerase Chain Reaction (RT-PCR)

Total RNA was isolated from HUVECs by using RNeasy mini kit. RT was performed using the RT system according to the manufacturer’s instructions. The PCR program was as follows: hold at 95°C for 5 min, then 95°C for 45s, 62°C for 45s and 72°C for 1 min for 37 cycles. The specific sense and antisense primers for human PPARγ included 5’-ttgcagtggggatgtctcat-3’ and 5’-tttcctgtcaagatcgccct-3’, and the expected size of PPARγ fragments was 208 bp. The specific sense and antisense primers for human glyceraldehyde-3-phosphate dehydrogenase (GAPDH) included 5’-gtcagtggtggacctgacct-3’ and 5’-tgctgtagccaaattcgttg-3’, and the expected size of GAPDH fragments was 245 bp.

### Transfection Experiments

SignalSilence Akt siRNA (Cell Signaling Technology, Inc. Danvers, MD, USA) or PPARγ siRNA (Santa Cruz Biotechnology, Santa Cruz, CA, USA) were transfected into HUVECs using Lipofectamine according to the protocol. Cells (40% confluent) were serum-starved for 1 h followed by incubation with 100 nM target siRNA or control siRNA for 6 h in serum-free media. The serum-containing media was then added (10% serum final concentration) for 42 h before experiments and/or functional assays were conducted. Target protein silencing was assessed through protein analysis up to 48 h after transfection.

### Measurement of Nitric Oxide (NO) Production

NO production was analyzed using a commercial NO Assay kit (Thermo Fisher Scientific Inc, Rockford, IL, USA) based on the Griess reaction according to the manufacturer’s instructions. Briefly, after treatment, accumulated nitrite (NO_2_^−^) and nitrate (NO_3_^−^), the stable breakdown product of NO in culture media was measured by mixing Griess reagent and then incubating at room temperature for 15 min. The azo dye production was then determined spectrophotometrically at 540 nm. Sodium nitrite was used as a standard. The amount of NO production was normalized against that of total cellular protein.

### Immunoblottings

After the different treatments, HUVECs were rinsed once with ice-cold PBS before addiction of the lysis buffer (100 mM Tris-HCl, pH6.8, 4%SDS, 20% glycerol, 1 mM sodium orthovanadate, 1 Mm NaF, and 1 mM phenylmethylsulfonylfluoride) to the dished on an ice try. The cell lysates were scraped, boiled, and centrifuged for 2 min at 13.000 rpm. Cell lysates were separated by SDS-PAGE. Antibodies used were: endothelial nitric oxide synthase (eNOS), phosphorylated eNOS at Ser1177, Akt, phosphorylated Akt at Ser473 (Cell Signaling Technology, Inc. Danvers, MD, USA). Primary and secondary antibodies were incubated with the membranes with standard technique. Immunodetection was accomplished using enhanced chemiluminescence.

### Cell Immunofluorescence

HUVECs were grown on coverslips and exposed to treatments. Cells were fixed with 4% paraformaldehyde for 30 min and permeabilized with 0.1% Triton X for 5 min. Blocking was performed with 3% normal serum for 20 min. Cells were incubated with antibody against tubulin (Santa Cruz) and FITC-conjugated secondary antibody (K00018968, Dako North America Inc., Dako, Denmark). After washing the nuclei were counterstained with 4'-6-diamidino-2-phenylindole (DAPI) (Sigma). The coverslips were mounted with Vectashield mounting medium (Vector Laboratories, Burlingame, CA). Immunofluorescence was visualized using an Olympus BX41 microscope and recorded with a high-resolution DP70 Olympus digital camera. Pictures were photographed.

### Statistical Analysis

Data are presented as means ± SD, representing at least 3 independent experiments. Statistical comparisons were made using Student’s t test or one-way analysis of variance followed by a post hoc analysis (Tukey test) where applicable to find means that are significantly different from each other, and the significance level was set at *P* < 0.05.
